# Hospitalization costs in Portugal among people with obesity: results from a nationwide population-based cohort 2011 to 2021

**DOI:** 10.3389/fpubh.2024.1380690

**Published:** 2024-04-24

**Authors:** Kelli Destri, Ana Rita Henriques, Nuno Mendonça, Joana Alves, Anabela Barcelos, Sara Simões Dias, Maria João Gregório, Helena Canhão, Ana Maria Rodrigues

**Affiliations:** ^1^Comprehensive Health Research Centre, NOVA Medical School, Universidade NOVA de Lisboa, Lisbon, Portugal; ^2^NOVA National School of Public Health, Public Health Research Centre, Comprehensive Health Research Center, NOVA University Lisbon, Lisbon, Portugal; ^3^Department of Rheumatology, Centro Hospitalar do Baixo Vouga, Aveiro, Portugal; ^4^Center for Innovative Care and Health, Polytechnic of Leiria, Leiria, Portugal; ^5^Programa Nacional Para a Promoção da Alimentação Saudável, Direção-Geral da Saúde, Lisbon, Portugal; ^6^Faculdade de Ciências da Nutrição e Alimentação, Universidade do Porto, Porto, Portugal

**Keywords:** obesity, economic, epidemiology, public health, hospitalizations

## Abstract

**Background:**

Obesity has been extensively studied over the years, primarily focusing on the physiological aspects of the disease. However, the general burden of obesity mainly the financial implications and its influence on hospitalization and length of stay have only recently garnered attention in the literature, particularly in the case of Portugal.

**Aim:**

This study aimed to investigate the association between obesity and hospitalizations in the Portuguese adult population and compare the average costs of hospitalization among participants with and without obesity.

**Methods:**

At baseline, the analytic sample consisted of 10,102 participants aged ≥18 years from the Portuguese population-based Epidemiology of Chronic Diseases Cohort (EpiDoC). Participants were then followed for up to 10 years from 2011 to 2021 in three more waves of data collection. Body mass index was derived from self-reported weight and height, and instances of hospitalization were self-reported by the participants. The associated costs for each hospitalization episode were categorized according to national legislation and valued according to the pricing for Diagnosis Related Groups.

**Results:**

Obesity was associated with more hospitalizations (for example, Obesity class I vs. normal weight: OR = 1.33 [1.14–1.55]). However, when the presence of multimorbidity was considered, this association diminished. While longer hospital length of stay was observed in individuals with higher obesity categories, this difference did not reach statistical significance. On average, the total hospitalization costs per patient with obesity amounted to €200.4 per year.

**Conclusion:**

Obesity is as a risk factor for hospitalizations and potentially with higher length of stay hospitalizations, with this effect being partially mediated by the concurrent presence of multimorbidity. Consequently, obesity constitutes an additional burden on healthcare systems. This underscores the imperative of implementing cost-effective prevention programs aimed at addressing and managing this significant public health concern.

## Introduction

In 2005, approximately 9.8% of the world’s adult population had obesity, this number continued to grow and in 2015 12% of the global population were obese (1). It is projected that by 2030, there will be a 33% increase globally in the prevalence of obesity, resulting in a total of 1.12 billion individuals affected by obesity, as compared to the statistics from 2005 (2, 3). As per the World Health Organization (WHO), nearly a quarter (23%) of adults within the European Region were afflicted by obesity, which was a higher prevalence than any other region except for the Americas. In the specific case of Portugal, the data from the first National Health Examination Survey (INSEF 2015) revealed that approximately 28% of the population was affected by obesity (4).

While the WHO recognizes obesity as a chronic disease, it is not typically identified as the primary cause of hospitalization in the global literature. Nonetheless, obesity serves as a significant risk factor for a multitude of other chronic conditions, including but not limited to chronic obstructive pulmonary disease, pneumonia, heart failure, acute myocardial infarction, and inflammatory bowel disease. These comorbidities can contribute to a deterioration in an individual’s health, necessitating more intricate treatment regimens that may ultimately lead to hospitalization (1, 5–7).

Previous research suggests that healthcare systems worldwide face substantial costs due to overweight and obesity. In the United States, obesity accounts for approximately 5.5–7.0% of health expenditures, while in other countries from 2.0–3.5%. Moreover, previous research have indicated that medical costs with obese individuals are 30% higher compared to those with normal weight. A review of evidence in the United Kingdom, found direct costs associated with overweight and obesity of £3.2 billion, with estimates ranging between £480 million in 1998 to £1.1 billion in 2004 (8–10).

The economic impact of obesity on hospitalization costs is quite heterogeneous in the literature, influenced by factors such as the scope of costs considered and the inclusion of different conditions and treatments.

For example, a study for Sweden, using regression models, estimated annual hospital costs of 1.36 billion for male obese individuals and 0.81 billion for female obese individuals (11). This represented 2.3% of Sweden’s total hospital care costs that year. Similarly, a study for Germany in 2002, using attributable fractions based on prevalence data and relative risks from United States, estimated direct costs attributable to obesity and overweight around 4,854 million euros, of which 21% were attributed to hospital stays (1,036 million of euros) (12). Another cost of illness study for Republic of Ireland, from 1997 to 2004, reported annual hospital costs ranging from 4.4 million euros in 1997 to 13.3 million euros in 2004 (13).

Moreover, a systematic review and meta-analysis, focusing on childhood overweight and obesity, found increased hospitalization costs for obese individuals ($2439.14; 95%CI: 2135.93, 2742.36) compared with the overweight ($142.27, 95%CI: −494.92, 779.47). Variations were observed based on the primary diagnosis. The increase in hospitalization costs was substantially higher for obesity-related primary diagnoses ($6997.29; 95%CI: 6864.40, 7130.18) than for other conditions such as appendicitis ($5503.95; 95%CI: 5370.67, 5637.22) or adenotonsillectomy $902.64 (95%CI: 497.19, 1308.08) (14).

Pereira et al. (15) employed data from the year 1996 to compute the direct costs with obesity in Portugal. These costs constituted 3.5% of the total healthcare expenses at that time. The study encompassed not only hospitalization, which accounted for 29% of the overall direct expenses related to obesity but also encompassed outpatient care, including consultations, medications, laboratory tests, and examinations. It is important to note, however, that the study was reliant on data from a relatively antiquated period, emphasizing the necessity for further up to date cost estimations. In Portugal, there were over 900,000 hospitalizations for various reasons in 2020 (16), with an average length of stay of 9.4 days, marking one of the lengthiest durations within the European Union (17). Inpatient costs constitute a significant portion of healthcare expenditures, accounting for 28% of the country’s total healthcare expenditures. Consequently, it is imperative to acquire further insights into the costs associated with hospitalizations among individuals with obesity.

The primary objective of this study is to evaluate the relationship between obesity and hospitalizations, while also providing an analysis of the duration of hospital stays and associated costs and comparing the average costs of hospitalization among participants with and without obesity. This analysis will be conducted using a representative sample of the Portuguese population.

## Methods

### Study design

The data analyzed in this study were collected as part of the Epidemiology of Chronic Diseases Cohort (EpiDoC), initiated in 2011. EpiDoC is a closed prospective cohort that aimed to create a large population database for medical and health-related research in Portugal. It comprises a representative sample of adults (≥18 years old) who were non-institutionalized and living in private households in mainland Portugal or islands (Azores and Madeira). Participants were selected using multistage random sampling, as described elsewhere (18).

The EpiDoC cohort had four waves: EpiDoC 1 (*N* = 10,661) collected baseline data from September 2011 to December 2013; EpiDoC 2 (*N* = 7,591) started in March 2013 and ended in July 2015; EpiDoC 3 (*N* = 5,653) started in September 2015 and ended in July 2016; and the most recent wave, EpiDoC 4 (*N* = 3,757), occurred from March to August 2021.

The baseline assessment involved a face-to-face interview, in the remaining waves data were collected via a structured questionnaire through phone interviews using a computer-assisted personal interview system.

### Study population

This study included participants from the EpiDoC cohort who provided self-reported information on hospitalization within the last 12 months, as well as data on their height and weight. The exclusion criteria included non-responses to the question “Have you been hospitalized in the last 12 months?” or answering “Does not know/ does not answer,” as well as without self-reported height and weight.

### Outcomes

#### Hospitalization

Hospitalizations were self-reported based on the question, “Have you been hospitalized in the last 12 months?” (Yes/No). This information was collected across all four waves of the study.

#### Hospital length of stay

The hospital length of stay (LOS) was determined by the number of days the individual was hospitalized, as reported in response to the question “How long were you hospitalized.” The hospital LOS was measured only during the follow-up waves (EpiDoC 2 to EpiDoC 4). Consequently only three time points were considered with the EpiDoc 2 serving as the baseline (18).

#### Costs of hospitalization

To calculate the costs associated with hospitalizations, we first calculate all of hospital admissions based on the 2018 Homogeneous Diagnosis Groups (DRG), as outlined in Portaria n° 254/2018 dated September 7th. This legislation not only detalis the pricing for each DRG but also categorize inpatient episodes in short, normal, or extended lengths of stay (LOS). Moreover, its sets the prices per day for patient within these categories (19). We used this specified price as a proxy for the costs associated with hospitalization, a common approach widely adopted in the existing literature (11, 20, 21). Given the absence of baseline data on the duration of hospitalizations in the EpiDoc cohort, we uniformly priced all hospital episodes as episodes of normal duration (17, 22).

After the initial analysis, average hospitalization costs were calculated among participants with and without obesity separately. Subsequently, an extrapolation of yearly hospitalization expenditures was derived for individuals with obesity and those without obesity, considering the prevalence of adults with and without obesity in the Portuguese population in 2019. These estimates were determined using the formula: total number of individuals with obesity multiplied by the average hospitalization costs for individuals with obesity and individuals without obesity multiplied by the average hospitalization costs for individuals without obesity.

### Exposure of interest

#### Body mass index

BMI was calculate using self-reported height and weight BMI = weight in kilograms / (height in meters)^2^. Participants were then categorized into the standard BMI classifications: underweight (BMI < 18.5), normal weight (BMI 18.5 to <25 kg/m^2^), overweight (BMI 25 to <30 kg/m^2^), Obesity class I (BMI 30 to <35 kg/m^2^), Obesity class II (BMI 35 to <40 kg/m^2^), and Obesity class III (BMI ≥ 40 kg/m) (23).

#### Covariates

Other variables were included such as demographic and socioeconomic variables, including age, sex, nomenclature of territorial units for statistics level 2 (NUTS 2) region (North, Center, Lisbon, Alentejo, Algarve, Azores, and Madeira), years of education, marital status (single, married, stable union, divorced and widowed). Smoking habits (never smoked, former and current smoker), alcohol intake (alcohol drinkers or not) and regular physical exercise (yes, no) were also collected. Multimorbidity was categorized as the presence of two or more chronic disease. This classification was determined by examining responses related to condition such as hypertension, high cholesterol, diabetes, respiratory, gastro, cardiac, neurological, mental, oncological, and rheumatic diseases (24).

#### Statistical analyses

Descriptive statistics for continuous variables include mean and standard deviations, while categorical variables were presented as counts and percentages. To assess the relationship between obesity and three distinct outcomes (prior hospitalization, length of hospital stay, and hospitalization costs), tailored statistical models were employed to account the specific distribution characteristics of each outcome variables.

To examine the association between obesity and a hospitalization event, mixed-effects logistic models, the relationship between obesity and length of hospital stay (LOS), we used zero-inflated negative binomial mixed-effects models that allow us to incorporate the well-developed analytic procedures into the framework for analyzing over-dispersed and zero-inflated count or proportion data with multilevel structures (e.g., longitudinal studies). In the case of hospitalization costs, we employed zero-inflated mixed-effects gamma models with a log link, due to the existence of zero costs for some patients. For each outcome under examination, we implemented multiple models with varying degrees of adjustment. Model 1, which included id (random effect), time since the start of the study, and obesity (fixed effect); Model 2, additionally adjusted for sex, age at baseline, NUTS 2 region, years of education, and employment status (fixed effects); Model 3, which further incorporated lifestyle factors, such as alcohol consumption, smoking habits, and regular exercise (fixed effects).

The average annual hospitalization cost among participanst with and without obesity were compared using a *t*-test.

A sensitive analysis was conducted to investigate the potential mediating role of multimorbidity ion the obesity-hospitalization relationship. Adjustment for multimorbidity was added in the final model assessing the associations between obesity and hospitalization (25–28).

As the number of underweight participants was very small (1.6%), this category was not considered in any of the models. Analyses were performed in STATA v.17 and R version 4.1.1, and *p* < 0.005 was considered statistically significant. Zero-inflated models were fitted using the R *glmmTMB* package (29).

## Results

### Baseline characteristics

At the outset of the study, a total of 10,102 participants were included in the analysis. Among these individuals, 862 (8.5%) had experienced hospitalization, with 228 (26.5%) of those hospitalized individuals being classified as having obesity. The average age of the participants was 52.8 years with a standard deviation of 18.0, and it was observed that hospitalized patients were slightly older in comparison to those who did not report any hospitalizations (55.8 years ±17.6 vs. 51.6 years ±17.7). In terms of gender distribution, there were more women among the participants who had been hospitalized compared to those who had not (64.6% vs. 60%). Furthermore, the presence of multimorbidity was significantly more common among the hospitalized participants (58.5% vs. 40.5%) (Table 1).

**Table 1:** Baseline sociodemographic and health characteristics of participants per hospitalization (yes/no).

	Total *n =* 10,102	Hospitalization (Yes) *n =* 862	Hospitalization (No) *n =* 9,240
**Sociodemographic**			
Women	6,101 (60.4%)	557 (64.6%)	5,544 (60.0%)
Age (mean ± sd)	52.0 ± 17.7	55.8 ± 17.6	51.6 ± 17.7
**Age group**			
18–25 years	811 (8.0%)	47 (5.5%)	764 (8.3%)
26–35 years	1,231 (12.2%)	97 (11.3%)	1,134 (12.3%)
36–45 years	1793 (17.8%)	106 (12.3%)	1,687 (18.3%)
46–55 years	1838 (18.2%)	152 (17.6%)	1,686 (18.3%)
56–65 years	1805 (17.9%)	163 (18.9%)	1,642 (17.8%)
66–75 years	1,586 (15.7%)	166 (19.3%)	1,420 (15.4%)
76–85 years	908 (9.0%)	114 (13.2%)	794 (9.0%)
>85 years	130 (1.3%)	17 (2.0%)	113 (1.2%)
**Marital status**			
Married	5,850 (58.0%)	513 (59.5%)	5,337 (57.8%)
Other	4,245 (42.0%)	349 (40.5%)	3,896 (42.2%)
**Education level**			
0–4 years	4,286 (42.7%)	430 (50.2%)	3,856 (42.0%)
5–9 years	2,122 (21.1%)	157 (18.3%)	1965 (21.4%)
10–12 years	1896 (18.9%)	148 (17.3%)	1748 (19.0%)
>12 years	1737 (17.3%)	122 (14.2%)	1,615 (17.6%)
**NUTS 2**			
Norte	2,972 (29.4%)	286 (33.2%)	2,686 (29.1%)
Centro	1846 (18.3%)	156 (18.1%)	1,690 (18.3%)
Lisboa	2,424 (24.0%)	223 (25.9%)	2,201 (23.8%)
Alentejo	583 (5.8%)	49 (5.7%)	534 (5.8%)
Algarve	325 (3.2%)	25 (2.9%)	300 (3.3%)
Azores	973 (9.6%)	83 (9.6%)	890 (9.6%)
Madeira	979 (9.7%)	40 (4.6%)	939 (10.2%)
**Employment status**			
Employed full-time/part-time	4,265 (42.5%)	238 (27.7%)	4,027 (43.9%)
Unemployed	1,048 (10.5%)	96 (11.2%)	952 (10.4%)
Retired	3,387 (33.8%)	374 (43.6%)	3,013 (32.8%)
Student/ Domestic worker/ Temporally work disabled	1,333 (13.3%)	150 (17.5%)	1,183 (12.9%)
**Lifestyle**			
BMI (kg/m^2^)			
Underweight	166 (1.6%)	7 (0.8%)	159 (1.7%)
Normal weight	4,069 (40.3%)	308 (35.7%)	3,761 (40.7%)
Overweight	3,798 (37.6%)	319 (37.0%)	3,479 (37.7%)
Obesity class I	1,542 (15.3%)	172 (20.0%)	1,370 (14.8%)
Obesity class II	403 (4.0%)	43 (5.0%)	360 (3.9%)
Obesity class III	124 (1.2%)	13 (1.5%)	111 (1.2%)
**Alcohol intake**			
Daily	1967 (19.5%)	176 (20.5%)	1791 (19.4%)
Occasional	3,845 (38.1%)	250 (29.1%)	3,595 (39.0%)
Never	4,274 (42.4%)	434 (50.5%)	3,840 (41.6%)
**Smoking habits**			
Daily	1806 (17.9%)	146 (16.9%)	1,660 (18.0%)
Occasionally	234 (2.3%)	10 (1.2%)	224 (2.4%)
In the past	1926 (19.1%)	208 (24.1%)	1718 (18.6%)
Never	6,136 (60.7%)	498 (57.8%)	5,638 (61.1%)
**Regular physical exercise**			
Yes	3,413 (33.8%)	231 (26.8%)	3,182 (34.5%)
**Health**			
Number of chronic noncommunicable diseases (self-reported)			
0–1	5,863 (58.0%)	358 (41.5%)	5,505 (59.6%)
≥2	4,239 (42.0%)	504 (58.5%)	3,735 (40.5%)
Chronic noncommunicable diseases (self-reported)			
High blood pressure	3,087 (30.8%)	331 (38.8%)	2,756 (30.0%)
Diabetes	1,106 (11.0%)	153 (17.9%)	953 (10.4%)
High cholesterol	3,120 (31.3%)	305 (36.1%)	2,815 (30.8%)
Pulmonary disease	583 (5.8%)	94 (11.0%)	489 (5.3%)
Cardiac disease	1,224 (12.2%)	199 (23.4%)	1,025 (11.2%)
Chronic noncommunicable diseases (self-reported)			
Gastrointestinal disease	1,693 (16.9%)	209 (24.6%)	1,484 (16.2%)
Neurological disease	373 (3.7%)	60 (7.0%)	313 (3.4%)
Mental disease	1,508 (15.0%)	186 (21.8%)	1,322 (14.4%)
Cancer	406 (4.0%)	98 (11.5%)	308 (3.4%)
Rheumatic disease	2,749 (28.1%)	327 (39.5%)	2,422 (27.0%)

NUTS 2, Nomenclature of territorial units for statistics level 2; BMI, Body Mass Index. Sample size is not constant due to missing values in some variables: **Total:** Sex (*n* = 10,102); Age (*n* = 10,102); Marital status (*n* = 10,095); Education level (*n* = 10,041); NUTS II (*n* = 10,102); Employment status (*n* = 10,033); BMI (*n* = 10,102); Alcohol intake (*n* = 10,086); Smoking Habits (*n* = 10,102); Regular physical exercise (*n* = 10,097); Number of chronic noncommunicable diseases (*n* = 10,102); High blood pressure (*n* = 10,038); Diabetes (*n* = 10,037); High cholesterol (*n* = 9,976); Pulmonary disease (*n* = 10,048); Cardiac disease (*n* = 10,021); Gastrointestinal disease (*n* = 10,026); Neurological disease (*n* = 10,047); Mental disease (*n* = 10,053); Cancer (*n* = 10,054); Rheumatic disease (*n* = 9,794). **Hospitalization (yes):** Sex (*n* = 862); Age (*n* = 862); Marital status (*n* = 862); Education level (*n* = 857); NUTS II (*n* = 862); Employment status (*n* = 858); BMI (*n* = 862); Alcohol intake (*n* = 860); Smoking habits (*n* = 862); Regular Physical Exercise (*n* = 861); Number of chronic noncommunicable diseases (*n* = 862); High blood pressure (*n* = 854); Diabetes (*n* = 856); High cholesterol (*n* = 844); Pulmonary disease (*n* = 856); Cardiac disease (*n* = 850); Gastrointestinal disease (*n* = 209); Neurological disease (*n* = 853); Mental disease (*n* = 853); Cancer (*n* = 851); Rheumatic disease (*n* = 827). **Hospitalization (No):** Sex (*n* = 9,240); Age (*n* = 9,240); Marital Status (*n* = 9,233); Education level (*n* = 9,184); NUTS II (*n* = 9,240); Employment status (*n* = 9,175); BMI (*n* = 9,240); Alcohol intake (*n* = 9,226); Smoking habits (*n* = 9,240); Regular Physical Exercise (*n* = 9,236); Number of chronic noncommunicable diseases (*n* = 9,240); High blood pressure (*n* = 9,184); Diabetes (*n* = 9,181); High cholesterol (*n* = 9,132); Pulmonary disease (*n* = 9,192); Cardiac disease (*n* = 9,171); Gastrointestinal disease (*n* = 9,177); Neurological disease (*n* = 9,194); Mental disease (*n* = 9,200); Cancer (*n* = 9,203); Rheumatic disease (*n* = 8,967).

Individuals classified as having obesity (class I, II, or III) exhibited a slightly higher percentage of hospitalizations compared to those with a normal weight in each wave, with statistically significant differences observed in the first two waves (*p* < 0.01). Similarly, the length of hospital stay (LOS) displayed a similar pattern, with slightly longer LOS for participants with obesity at EpiDoC 1 (*p =* 0.05), EpiDoC 2 (*p =* 0.21), and EpiDoC 3 (*p =* 0.41), although statistical significance was only reached in the first wave (Table 2).

**Table 2:** Characteristics of hospitalizations stratified by body mass index (BMI) categories.

BMI category	Total	Underweight	Normal weight	Overweight	Obesity class I	Obesity class II	Obesity class III	
EpiDoC 12,011–2013	(*n =* 10,102)	(*n =* 166)	(*n =* 4,069)	(*n =* 3,798)	(*n =* 1,542)	(*n =* 403)	(*n =* 124)	*p*-value
Hospitalization (yes)	862 (8.5%)	7 (4.2%)	308 (7.6%)	319 (8.4%)	172 (11.2%)	43 (10.7%)	13 (10.5%)	<0.001^a^
Length of stay (days) (mea*n* ± sd)	–	–		–	–	–	–	
EpiDoC 22,013–2015	(*n =* 6,905)	(*n =* 111)	(*n =* 2,666)	(*n =* 2,779)	(*n =* 1,017)	(*n = 2*50)	(*n =* 82)	*p*-value
Hospitalization (yes)	590 (8.5%)	8 (7.2%)	172 (6.5)	259 (9.3%)	109 (10.7%)	28 (11.2%)	14 (17.1%)	<0.001^a^
Length of stay (days) (mea*n* ± sd)	9.3 ± 17.4	5.3 ± 3.1	9.8 ± 15.0	9.0 ± 19.4	10.5 ± 19.8	6.1 ± 6.1	8.2 ± 8.2	0.05^b^
EpiDoC 32,015–2016	(*n =* 5,159)	(*n =* 88)	(*n =* 2003)	(*n =* 2091)	(*n =* 745)	(*n =* 181)	(*n =* 51)	*p*-value
Hospitalization (yes)	683 (13.2%)	234 (11.2%)	225 (11.2%)	278 (13.3%)	123 (16.5%)	37 (20.4%)	11 (21.6%)	0.149^a^
Length of stay (days) (mea*n* ± sd)	8.5 ± 16.3	11.6 ± 20.4	8.3 ± 14.5	7.9 ± 15.0	7.7 ± 18.7	15.5 ± 23.0	10 ± 13.5	0.21^b^
EpiDoC 42,021	(*n =* 3,415)	(*n =* 39)	(*n =* 1,241)	(*n =* 1,384)	(*n =* 567)	(*n =* 143)	(*n =* 41)	*p*-value
Hospitalization (yes)	332 (9.7%)	4 (10.3%)	111 (8.9%)	123 (8.9%)	71 (12.5%)	18 (12.6%)	5 (12.2%)	0.472^a^
Length of stay (days) (mea*n* ± sd)	6.2 ± 11.2	6.5 ± 8.5	5.5 ± 5.5	6.1 ± 10.0	7.4 ± 15.3	5.3 ± 4.9	8.2 ± 5.1	0.41^b^

^a^Chi-square test. ^b^Kruskal Wallis test. Information on length of stay was not collected at EpiDoC 1.

### Hospitalizations and obesity

**Figure 1:**
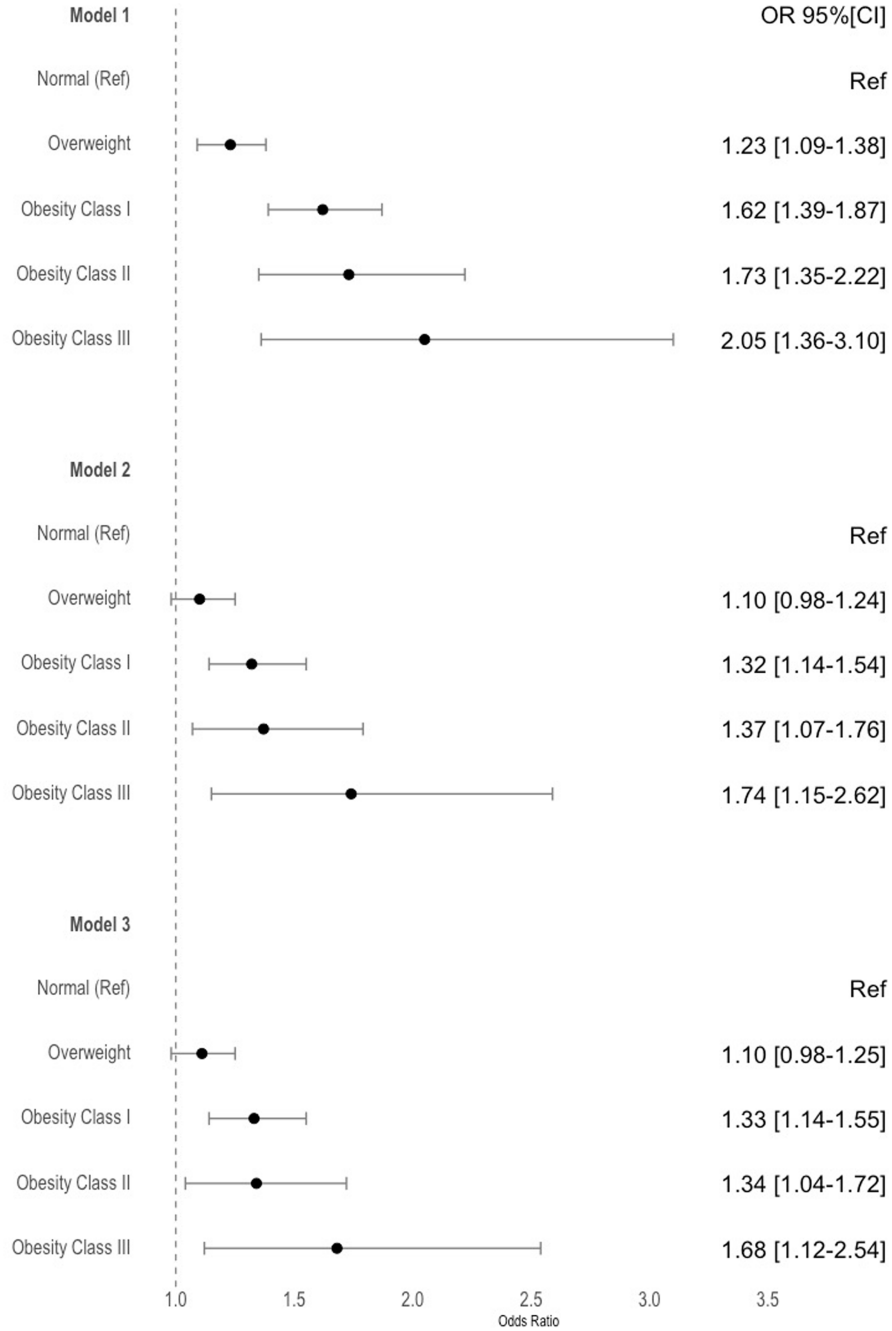
Estimates and 95% confidence intervals for the odd of being hospitalized over time per obesity categories. Model 1 was adjusted for time; Model 2 was further adjusted for sex, age, NUTS 2, employment status and education level; Model 3 was further adjusted for lifestyle factors; Normal weight was used as reference for all models.

In comparison to individuals with normal weight as a reference group, our analysis revealed a statistically significant association between obesity and the likelihood of hospitalization in all three models (Figure 1). The odds of hospitalization increased with higher obesity categories. In model 1, where adjustment was made solely for the number of years from baseline, this association was significant. This increasing relationship persisted in model 2 and model 3 after further adjustments were made. In the more completed model (model 3) odds ratios (OR) for hospitalization were as follows: Obesity class I: OR *=* 1.33 [1.14–1.55], Obesity class II: OR *=* 1.34 [1.04–1.72], Obesity class III: OR *=* 1.68 [1.12–2.54]. Model 2 included adjustments for sex, age, NUTS 2 region, employment status, education level, while model 3 encompassed all previous adjustments and was further adjusted for lifestyle factors (alcohol consumption, smoking habits, and regular exercise) (Figure 1).

**Figure 2:**
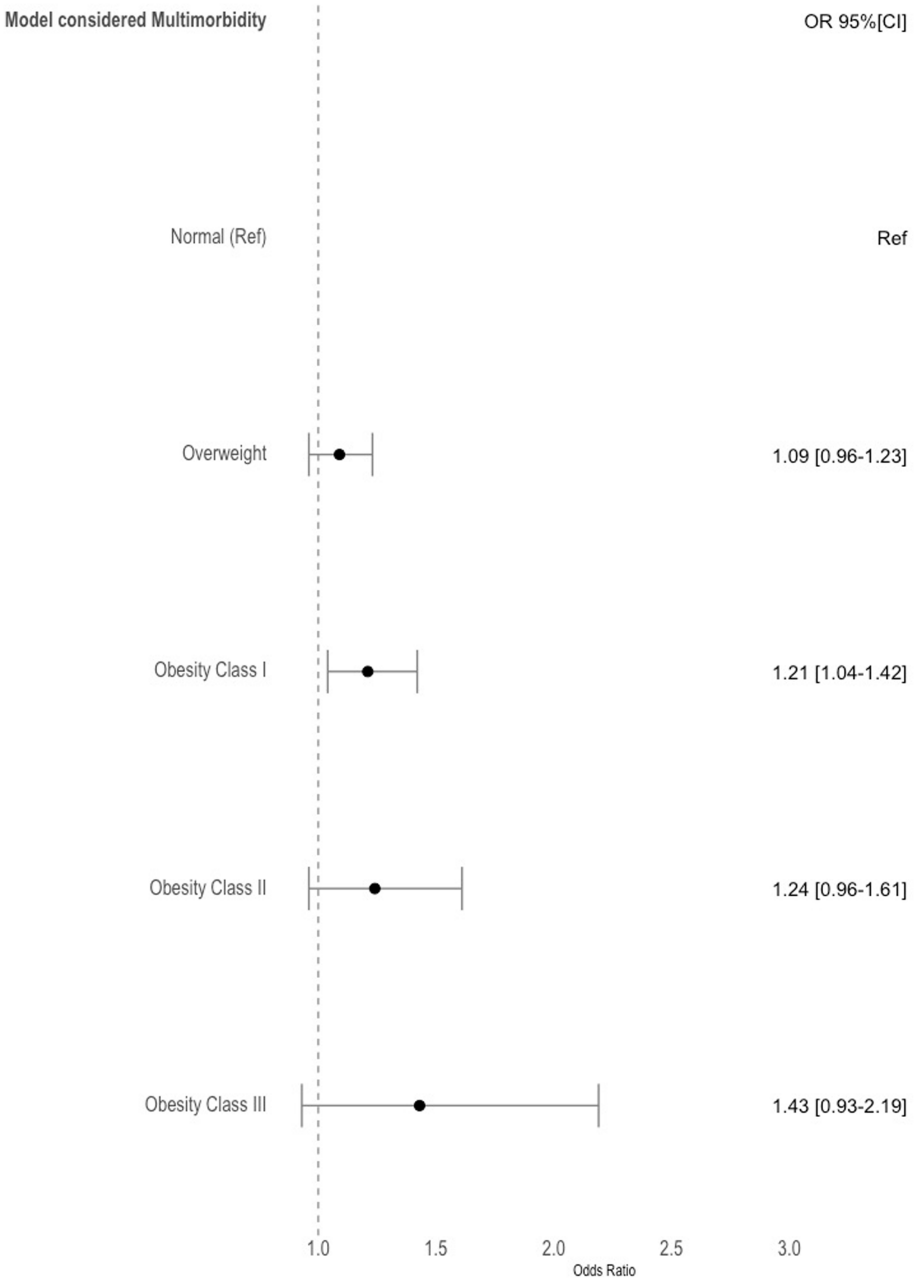
Estimates and 95% confidence intervals for the odds of being hospitalized over time per obesity categories. Model was adjusted for time, sex, age, NUTS 2, employment status, education level, alcohol consumption, smoking habits, regular exercise and multimorbidity. Normal weight was used as reference.

In our sensitivity analysis, where we considered the potential mediating role of multimorbidity in the relationship between obesity and hospitalization, we observed that the gradient between obesity and the odds of hospitalization persisted. However, it no longer reached statistical significance when adjusting for multimorbidity (Figure 2). This suggests that multimorbidity is likely to act as a partial mediator in the association between obesity and hospitalizations, indicating that the presence of multiple chronic conditions may contribute to the increased odds of hospitalization in individuals with obesity (25–28).

### Hospitalizations’ length of stay and obesity

**Figure 3:**
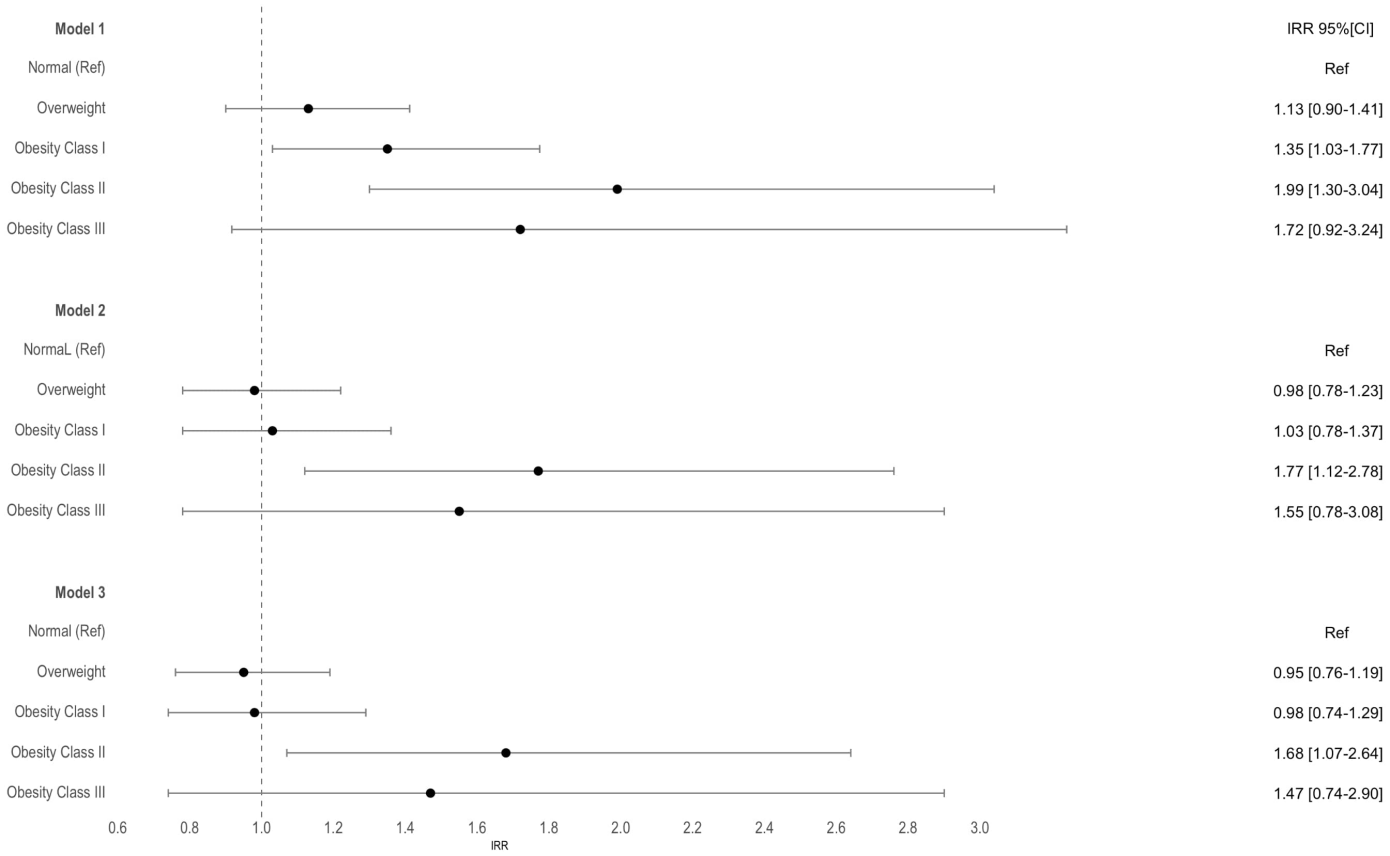
Incidence rate ratios (IRR) and 95% confidence intervals for the hospitalization length of stay per obesity categories. Model 1 was adjusted for time; Model 2 was further adjusted for sex, age, NUTS 2, employment status and education level; Model 3 was further adjusted for lifestyles; Normal weight was used as reference for all models.

In our analysis, we identified a statistically significant association between length of hospital stay (LOS) and obesity. In model 1, the incidence rate ratio (IRR) for LOS was Obesity class I: IRR *=* 1.35 [1.03–1.77], Obesity class II: IRR *=* 1.99 [1.03–3.04]. This association with LOS was observed over time. However, after adjusting for demographic and lifestyle factors in models 2 and 3, the significant association remained only for individuals in obesity class II, with an IRR of 1.77 [1.12–2.70] in model 2 and an IRR of 1.68 [1.07–2.60] in model 3. No significant association was observed for obesity class I or III (Figure 3). This suggests that obesity class II is independently associated with a longer length of hospital stay, even after considering demographic and lifestyle factors, while class I and III obesity did not exhibit a statistically significant association with LOS after such adjustments.

### Hospitalizations costs and obesity

The average annual hospitalization cost among participants with obesity was €200.4 ± €824.7. Specifically, for obesity class I, it was €194.6 ± €830.4, for obesity class II, it was €211.6 ± €795.0, and for obesity class III, it was €238.6 ± €847.8. In contrast, the hospitalization cost among non-obese individuals was €136.9. On average, hospitalization costs for participants without obesity were €63.5 lower compared to hospitalizations for participants with obesity, and this difference was statistically significant (*p* = 0.001).

Given the 17.7%, prevalence of obesity among the Portuguese population in 2019, the estimated annual hospitalization costs for people with obesity would be approximately €365 million. Likewise, for individuals without obesity, considering a prevalence of 43.4% in the Portuguese adult population, the estimated annual hospitalization costs for people without obesity would be around €182 million. Consequently, it can be inferred that there is an additional expenditure of €183 million per year associated with obesity when comparing the hospitalization costs of those with obesity to those without obesity (30).

**Figure 4:**
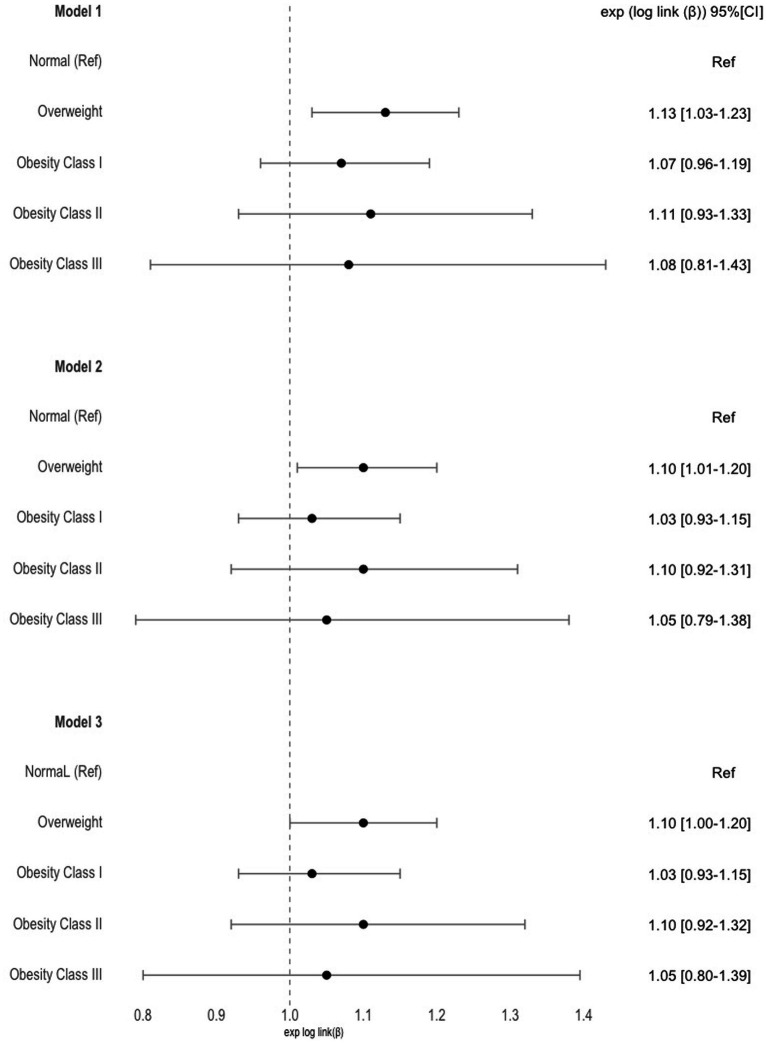
Estimates and 95% confidence intervals for the costs of hospitalization over time per obesity categories. Model 1 was adjusted for time; Model 2 was further adjusted for sex, age, NUTS 2, employment status and education level; Model 3 was further adjusted for lifestyle factors; Normal weight was used as reference for all models.

In the analysis concerning the relationship between hospitalization costs and various classes of obesity, no statistically significant differences were observed when comparing participants with obesity to those with a normal weight. This suggests that, in terms of hospitalization costs, there was no significant variation among different classes of obesity when compared to individuals with a normal weight (Figure 4).

## Discussion

This study attempted to assess the correlation between obesity and hospitalization, along with its implications in terms of length of stay (LOS) and costs for individuals with obesity in Portugal. Our findings revealed increasing odds of hospitalization with higher levels of obesity. The results further indicated that multimorbidity acts as a partial mediator in the relationship between obesity and hospitalizations. It is crucial to underscore that obesity constitutes a risk factor for various chronic diseases, including cardiovascular disease, chronic obstructive pulmonary disease, diabetes, and hypertension. As elucidated by Agborsangaya et al. (25) and corroborated by Dong et al. (28), individuals with obesity are twice as likely to report multimorbidity compared to their non-obese counterparts. Additionally, those with multimorbidity experience more frequent hospitalizations and typically undergo longer hospital stays. Consequently, the coexistence of both multimorbidity and obesity exerts a cumulative effect on hospitalizations, heightening the risk of metabolic decompensation and exacerbating the resource utilization (25, 28).

The findings indicate that, in comparison to individuals with normal weight, those with obesity exhibit a heightened odds of hospitalization, an extended length of stay, and increased inpatient costs. Nevertheless, these associations were observed to be partially attenuated by other factors, as evidenced by a decrease in significance or importance when controlling for additional variables. For instance, in the context of controlling for multimorbidity, the inclusion of variables that lie along the causal pathway of the disease might diminish the impact of obesity on the ultimate outcome. This, in turn, could lead to a misinterpretation that obesity is not a substantial risk factor for the hospitalized population (24, 26, 30, 31).

The connection between obesity and hospitalization in the global literature is consistently characterized by the presence of other comorbidities. In other words, the majority of studies do not isolate obesity as the primary exposure variable for assessing the likelihood of hospitalization. Instead, these investigations often focus on obesity-related conditions, such as diabetes, high blood pressure, and cardiovascular diseases. In such cases, obesity is viewed as a mediator between these diseases and the ultimate outcome of hospitalization (5, 28, 32, 33).

In Portugal, a study conducted by Sarmento et al. (34) revealed that obesity accounted for a hospitalization rate of 3.33 per 10,000 inhabitants in 2017. Nevertheless, diseases associated with obesity, such as diabetes, hypertension, and heart failure, exhibited slightly higher hospitalization rates (6.83, 8.50, and 32.66%, respectively) (34). Remarkably, our results aligns with these findings, depicting a hospitalization rate of 27% among obese individuals. Additionally, similar or higher rates were observed for other non-communicable diseases such as heart disease (25%), hypercholesterolemia (37.3%), and hypertension (40%). These results highlight a substantial hospitalization rate that surpasses that of obesity alone for conditions like hypertension, diabetes, and various prevalent non-communicable chronic diseases, emphasizing once more the significance of comorbidities in the association between hospitalizations and obesity.

The concurrent presence of obesity and other chronic diseases significantly contributes to the frequency of hospitalization, a phenomenon consistently underscored by numerous authors and affirmed by our study. Our investigation identifies multimorbidity as a mediating factor between obesity and hospitalization, emphasizing that obesity represents a substantial burden for public health systems. This impact extends beyond the direct influence of obesity on hospitalization rates, highlighting that the condition serves as a catalyst for multiple other chronic diseases, thereby indirectly amplifying the demands placed on healthcare resources (5, 24–26, 28, 32).

The correlation between obesity and both length of stay (LOS) and costs did not exhibit significance when compared to individuals with normal weight. Notably, only in the case of obesity class II did the length of stay remain statistically significant after accounting for all adjustments. A comparable study observed a prolonged length of stay in obese individuals compared to those with normal weight, aligning with our findings. However, it is noteworthy that this same study identified a positive association between obesity and costs, a disparity from our study’s results (35). Hauck et al. (36) discovered that obese patients experience, on average, a hospitalization duration 1.8 days longer than individuals with normal weight. Additionally, Maradit Kremers et al. (35)emphasized a J-shaped relationship, indicating that the longest hospitalizations occur in patients at the extremes of the BMI spectrum. This observation aligns partially with our study’s outcomes (35, 36). Similar trends were identified by Shaffer (31) and Zizza et al. (37), where in obese individuals exhibited prolonged hospitalization times compared to those with normal weight. Notably, this significance persisted across various types of adjustments, establishing a causal relationship between obesity and length of stay (LOS) (31, 37).

Concerning costs, our study determined that the average cost of hospitalization among obese individuals was €200.4 per person. Notably, this cost exhibited an upward trajectory with increasing BMI classes; however, these findings were not statistical significant when compared to individuals with normal weight. Nevertheless, the estimate of annual costs associated with hospitalizations among individuals with obesity amounted to a substantial figure, reaching €365 million annuall, an additional expenditure of €183 million compared with the estimated costs associated with hospitalizations among participants without obesity. It is worth noting, in the context of National Accounts data from Statistics Portugal (2020), that the total inpatient costs in Portuguese public and private hospitals totalled €3.3 billion in 2019 (38). Consequently, the costs associated with obesity accounted for 11% of the total inpatient costs in that year. This aligns with findings reported by other authors, underscoring the substantial burden of costs attributable to obesity (3, 30, 39, 40). Obesity presents a significant threat to the financial sustainability of both healthcare systems and individual households. The expenses associated with treating patients with chronic diseases linked to high BMI, such as diabetes, cancer, and cardiovascular diseases, represent USD PPP 425 billion annually in OECD countries, G20, and EU28 countries (41).

In a systematic review conducted by von Lengerke et al. (42), all included studies reported excess costs associated with obesity. Nationally, the proportion of total healthcare costs attributable to obesity ranged between 2.1 and 4.7%. Additionally, according to one cohort study, this figure was estimated to be between 1.9 and 3.6% (42). In another study conducted in Brazil, it was found that hospitalization expenditures constituted the majority of costs for health systems, comprising approximately 68% of the total costs, amounting to US$ 4.5 billion per year (39, 42). On the contrary, Vellinga (13) estimated that the annual hospital cost of hospitalization in Ireland was €4.4 million in 1997, escalating to €13.3 million in 2004. This highlights the substantial variation in the cost of hospitalization, influenced by factors such as location and the methodologies employed in cost estimations. However, direct comparisons of these values should be approached with caution due to potential disparities in methodological approaches, including the consideration of obesity as a single risk factor or an obesity-related comorbidity, BMI classification, statistical methods used, economic approaches employed for cost estimation, currency and purchasing power differences, and population size. These variations can substantially affect outcomes and may lead to misinterpretations. Nonetheless, the focus on the wide agreement concerning the economic strain caused by increasing obesity rates worldwide, as underscored by the World Bank’s 2020 report, highlights a critical issue. Despite differences in methodology, the prevailing message is unequivocal: the expenses related to healthcare and reduced productivity due to obesity and its associated comorbidities are surging globally, impacting economies at every level of development. This common concern emphasizes the importance of making obesity prevention a priority within public health policies and fiscal strategies, with the goal of diminishing its socio-economic effects universally (43).

## Limitations

This study is subject to several limitations. Primarily, the BMI values relied on self-reported weight and height, introducing the potential for systematic bias (44). Secondly, the study is limited by the relatively small number of individuals who reported having been hospitalized (*n =* 923) in comparison to the total cohort size (*n =* 10,661). Consequently, the final sample size, after classifying BMI, may have been insufficient to robustly establish a relationship between the exposure variable and the primary outcomes over time. Additionally, it is important to note that baseline data on the length of hospital stays were not collected. To mitigate the absence of specific cost information in this wave, we used the cost for a normal inpatient case as a proxy. Nonetheless, this approach could potentially have influenced the accuracy of cost calculations.

## Conclusion

In conclusion, our study shows that Portuguese adults with obesity have higher odds of hospitalization compared to those with normal weight. The presence of multimorbidity appears to partially mediate the relationship between obesity and hospitalizations. Notably, the costs associated with hospitalizations for individuals with obesity amount to €365 million per year. Recognizing obesity as a secondary diagnosis is not only essential for evaluating increased expenses for the National Health System but also presents an opportunity for intervention and counseling during hospitalization and post-discharge. This comprehensive approach can contribute to reducing the burden of obesity across various fronts.

In future studies it is important to analyze the specific diseases and conditions that contribute most significantly to hospitalization rates and costs among individuals with obesity, explore how different combinations of comorbidities affect hospitalization rates and costs among individuals with obesity, and investigate disparities in obesity-related hospitalizations across different vulnerable groups.

## Data availability statement

The codebook and analytic code are available pending request from the authors while the dataset is available pending application and approval by the EpiDoC Coordinator - Ana Rodrigues (ana.m.rodrigues@nms.unl.pt).

## Ethics statement

The studies involving humans were approved by the National Committee for Data Protection and by the Ethics Committee of the Faculty of Medical Sciences of the Universidade NOVA de under the registration number 07–2011-CEFCM and 05–2012-CEFCM. The studies were conducted in accordance with the local legislation and institutional requirements. The participants provided their written informed consent to participate in this study. Written informed consent was obtained from the individual(s) for the publication of any potentially identifiable images or data included in this article.

## Author contributions

KD: Formal analysis, Investigation, Methodology, Software, Supervision, Validation, Visualization, Writing – original draft, Writing – review & editing. AH: Formal analysis, Methodology, Software, Validation, Visualization, Writing – review & editing. NM: Methodology, Supervision, Validation, Visualization, Writing – review & editing. JA: Methodology, Supervision, Validation, Visualization, Writing – review & editing. AB: Methodology, Supervision, Validation, Visualization, Writing – review & editing. SD: Conceptualization, Formal analysis, Investigation, Methodology, Software, Supervision, Validation, Visualization, Writing – review & editing. MG: Conceptualization, Investigation, Supervision, Validation, Visualization, Writing – review & editing. HC: Conceptualization, Funding acquisition, Investigation, Project administration, Resources, Supervision, Validation, Visualization, Writing – review & editing. AR: Conceptualization, Data curation, Funding acquisition, Investigation, Methodology, Project administration, Resources, Supervision, Validation, Visualization, Writing – review & editing.
